# Mechanism-Enhanced Active Attapulgite-Supported Nanoscale Zero-Valent Iron for Efficient Removal of Pb^2+^ from Aqueous Solution

**DOI:** 10.3390/nano12091591

**Published:** 2022-05-07

**Authors:** Liang Dai, Kai Meng, Weifan Zhao, Tao Han, Zhenle Lei, Gui Ma, Xia Tian, Jun Ren

**Affiliations:** 1 School of Environmental and Municipal Engineering, Lanzhou Jiaotong University, Lanzhou 730070, China; 12201085@stu.lzjtu.edu.cn (K.M.); 0618138@stu.lzjtu.edu.cn (W.Z.); 0619068@stu.lzjtu.edu.cn (T.H.); 12211055@stu.lzjtu.edu.cn (Z.L.); renjun@mail.lzjtu.cn (J.R.); 2 Key Laboratory of Yellow River Water Environment in Gansu Province, Lanzhou Jiaotong University, Lanzhou 730070, China; 3 College of Chemistry and Chemical Engineering, Ningxia Normal University, Guyuan 756000, China; 82007056@nxnu.edu.cn (G.M.); 82013021@nxnu.edu.cn (X.T.)

**Keywords:** attapulgite, nanometer zero-valent iron, Pb^2+^ removal, adsorption mechanism

## Abstract

In this study, attapulgite-supported nano zero-valent iron (nZVI@ATP) was synthesized by a liquid-phase reduction method using active attapulgite (ATP) as raw material, and used for Pb^2+^ remediation in aqueous solution. To understand the mechanism of Pb^2+^ removal, various techniques were used to characterize nZVI@ATP. The results showed that spherical nZVI particles were uniformly dispersed on the surface of ATP, and the agglomeration of nZVI particles was significantly weakened. The adsorption performance of nZVI@ATP for Pb^2+^ was greatly improved compared with that of ATP ore, in which the Fe/ATP mass ratio of 1:2 was the best loading ratio. Under the conditions of a temperature of 25 °C and a pH of 5.00, the initial concentration of Pb^2+^ was 700 mg/L, and the Pb^2+^ removal rate of nZVI@ATP was 84.47%. The adsorption of nZVI@ATP to Pb^2+^ was mainly a spontaneous endothermic reaction of heterogeneous surfaces, and the adsorption rate of nZVI@ATP to Pb^2+^ was proportional to pH in the range of 2–5.5. The presence of Na^+^, Mg^2+^, and Ca^2+^ can inhibit the removal of Pb^2+^, and Ca^2+^ has the strongest inhibition effect on the removal of Pb^2+^. The removal mechanism of Pb^2+^ by nZVI@ATP obtained from SEM-EDS, BET, XRD, FTIR and XPS included reduction, precipitation, and the formation of complexes.

## 1. Introduction

With the rapid increase in China’s industrial production, a large number of heavy metal elements are entering the water through the electroplating, mining, metallurgy, leather, and industries, resulting in their content exceeding the standard, seriously polluting China’s water environment. In addition, human activities such as the use of pesticides and fertilizers, domestic garbage disposal, and vehicle transportation also directly or indirectly increase the heavy metal content in water bodies [1]. Lead (Pb) is one of the most toxic industrial metals, and is used in making lead-based coatings, painting products, pigments, solder, armor, insecticides, and lead–acid batteries [2,3]. The main sources of Pb pollution are lead dust, lead slag, and lead-containing wastewater produced by lead mining, battery manufacturing, metal smelting, and oil painting industries [4]. Pb in the environment is difficult to degrade, and once it enters the environment, will result in long-term toxicity, presenting a potential threat to humans, as it can cause serious harm to the central nervous system, immune system, blood system, and skeletal system after entering the human body [5].

In recent decades, nanoscale zero-valent iron (nZVI) has been found to be a promising approach for heavy metal remediation [6]. Among the various approaches, nZVI stands out due to its unique structure and properties, and has become one of the most widely used nanomaterials in the environment, as well as currently being the most studied nanotechnology [7,8]. nZVI is prone to corrosion, and eventually has a “core–shell” structure with a dense Fe^0^ core in the inner layer and a thin amorphous shell in the outer layer. The shell is thought to be a mixed Fe^2+^/Fe^3+^ oxide phase, which is formed due to the spontaneous oxidation of Fe^0^ in the environment [9,10]. Nanoscale zero-valent iron (nZVI) has high surface energy and reactivity, and the physicochemical properties of nZVI and its reducing ability enable it to be applied to the rapid decontamination of many water-phase pollutants [11,12]. However, the removal effect of nZVI in the practical treatment of heavy metal pollution is not ideal, which can be attributed to the following points: (1) agglomeration: due to the existence of high surface energy and a strong internal magnetic effect, the agglomeration of nZVI particles is serious, which reduces the mobility of nZVI particles in water and the effective contact area with pollutants [13]; (2) easy aging: nZVI is highly reactive, and its structure and chemical properties will change over time in the natural environment, gradually corroding even in the anaerobic water environment [14]; (3) poor electron selectivity: as a strong reducing agent, nZVI acts with the target pollutant, and most of the electrons are not effectively transmitted to the reaction group, but rather to the hydrogen ions or dissolved oxygen in the aqueous phase, degrading its reducibility, and resulting in a reduction of effective electron utilization [15]. To avoid the agglomeration of nZVI particles and to improve the stability and mobility of nZVI, various modifications have been carried out, including by means of the surface modification method, the metal doping method, and the solid loading method.

Effective application of nZVI requires an appropriate support medium for fixation and dispersion. For this purpose, materials with high surface-to-volume ratios, such as activated carbon, graphite, and clay, have been extensively used in the field of pollution remediation as nZVI support materials [16,17,18]. ATP is a kind of hydrated magnesium-rich silicate clay mineral; it has a specific crystal structure and special surface charge distribution characteristics, and has strong adsorption and ion exchange performance [19]. Natural ATP has broad application prospects in the treatment of heavy metal pollution in water because of its rich mineral resources, easy access, low cost, non-toxicity, and wide application range [20]. ATP has a specific needle structure, good dispersion ability, and high mechanical properties, which can be used as an excellent solid negative carrier. Loading nZVI on ATP is expected to disperse nZVI particles, increase the contact area with heavy metals, and solve the problem of easy agglomeration of nZVI. Based on the understanding of attapulgite’s unique structure and adsorption properties, scholars at home and abroad have carried out in-depth research on attapulgite as a cheap natural adsorbent material. Yin et al. applied ATP and sepiolite to remove Pb^2+^, and the removal rate of Pb^2+^ by ATP was 81.5% [21]. Xu et al. studied the adsorption of Pb^2+^ and Cu^2+^ in aqueous solution before and after amino-modified ATP, and the adsorption results showed that the adsorption capacity of Pb^2+^ by modified composite material was superior to that of Cu^2+^ in unary and binary systems [22]. Huang et al. removed Pb^2+^ from the water after purification, high-temperature calcination, and hydrothermal loading of ATP, the saturated adsorption capacity of Pb^2+^ by the three modified ATP was 43.5, 53.9, and 127.6 mg/g, respectively [23]. The results show that ATP is an excellent adsorbent for water treatment, especially when loaded with nZVI [24,25].

In this study, low-cost Gansu ATP was selected as the carrier, and ATP was acid-treated and supported with zero-valent iron nanoparticles using the liquid-phase reduction method. The influence of factors such as pH value, coexisting cation, and temperature change on the adsorption of Pb^2+^ by nZVI@ATP was studied. The adsorption kinetics model, isothermal adsorption model, and the Van ‘t Hoff equation were used to explore the adsorption performance of nZVI@ATP on Pb^2+^ in water, and the adsorption mechanism was explored in combination with the changes of the apparent morphology, elemental composition, phase composition, and functional groups of the modified materials before and after adsorption.

## 2. Materials and Methods

### 2.1. Materials

Anhydrous ethanol (C_2_H_5_OH, ≥99.7%), ferrous sulfate heptahydrate (FeSO_4_·7H_2_O, ≥99.0%), nitric acid (HNO_3_), lead nitrate (Pb(NO_3_)_2_, ≥99.0%), sodium borohydride(NaBH4, ≥98.0%), sodium hydroxide (NaOH, ≥96.0%) and hydrochloric acid (HCl) were all purchased from Sinopharm Chemical Reagents Co., Ltd. (Shanghai, China). Sodium nitrate (NaNO_3_, ≥99.0%) was purchased from Tianjin Damao Chemical Reagent Factory (Tianjin, China). ATP raw ore used in this experiment came from Gansu Hanxing Environmental Protection Technology Co., Ltd. (Lanzhou, China). After crushing and grinding the ATP raw ore, pass through a 200-mesh sieve to obtain ATP powder, which is bagged for later use.

### 2.2. Preparation of nZVI@ATP

ATP was treated with 3 mol/L hydrochloric acid before use, stirred for 3 h at 1000 r/min with an electric stirrer at room temperature, and then treated with ultrasonic wave for 1 h. After standing for some time, the supernatant was dumped, and the lower sediment was washed with ultra-pure water until it was neutral. Finally, it was dried at 80 °C in the oven and ground through a 200-mesh sieve to obtain the pretreated ATP, which was sealed and stored for later use.

nZVI@ATP was prepared by the liquid-phase reduction method. It was synthesized by dissolving 27.802 g FeSO_4_·7H_2_O in 100 mL of deionized water and a certain amount of pickling ATP was added according to the mass ratio of ferric soil 3:1, 2:1, 1:1, 1:2, and 1:3, respectively. The mixture was stirred for 2 h under the protection of N_2_ to make it fully mixed, then 100 mL anhydrous ethanol was added and stirred for 30 min. NaBH_4_ solution was then dropped and stirred for 15 min after dropping to ensure the full reaction of NaBH_4_ and Fe^2+^. All the above reaction processes were carried out in an N_2_ atmosphere. Then, the solution was centrifuged, the supernatant was discarded and cleaned with anaerobic deionized water and anaerobic anhydrous ethanol successively and separated by centrifuge. The black solid obtained was dried in a vacuum drying oven and sifted through 200 mesh to obtain nZVI@ATP, which was vacuum-sealed and stored in the freezer layer for use. ATP does not need to be added in the preparation of pure nZVI, and the other steps are the same as nZVI@ATP. Black solid particles of nZVI are produced after reduction according to the following reaction.

(1) $${\mathrm{Fe}}^{2+}+2{\mathrm{BH}}_{4}^{-}+6H_{2}O\to {\mathrm{Fe}}^{0}+2B{\left(\mathrm{OH}\right)}_{3}+7H_{2}↑$$

### 2.3. Characterization of Adsorbents

The structure and surface morphology of ATP and nZVI@ATP before and after adsorption were analyzed by scanning electron microscopy (SEM) (GeminiSEM500, Carl Zeiss AG, Jena, Germany) and X-ray energy spectroscopy (EDS) (X-max ExtremeX, Oxford Instruments, Abingdon, UK). The FTIR spectra of ATP and nZVI@ATP were collected by Fourier-transform infrared spectroscopy (FTIR) (Vertex70, Bruker, Karlsruhe, Germany). FTIR samples were prepared by KBr compression method (1–2 mg sample, 100–120 mg KBr). Both samples and KBr were dried and ground to particle size less than 2 μm. Thin slices were prepared by hydraulic press with scanning wave number ranging from 4000 to 400 cm^−1^. X-ray diffraction (XRD) (Rigaku, Rigaku Corporation, Tokyo, Japan) was used to analyze the crystal structure and composition of the samples. XRD radiation type is Cu-Kɑ, wavelength is 0.15416 nm, scanning range is (−3)–145°. Zeta potential values of acid-washed ATP and nZVI@ATP were measured by Zeta potential tester (Zeta) (Nano-ZS90, Malvern Company, Malvern, UK). The surface chemistry of samples was carried out using an X-ray photoelectron spectroscopy (XPS) (Kratos AXIS Ultra DLD, Kratos, Manchester, UK). All XPS spectra were calibrated (C1s = 284.4 eV) and compared with the NIST X-ray photoelectron spectroscopy database (https://srdata.nist.gov/xps/Default.aspx, accessed on 9 April 2022). The specific surface areas and pore size of the adsorbents were analyzed by a specific surface area and porosity analyzer (BET) (ASAP2020, Micromeritics Instruments, Norcross, GA, USA) using the Barret-Joyner-Halenda (BJH) adsorption model and the t-plot micropore model.

### 2.4. Batch Experiments

Pb^2+^ removal by different materials was examined by adding 0.03 g ATP, nZVI, and nZVI@ATP with different mass ratios in 30 mL Pb^2+^ solution (700 mg/L), respectively. All heavy metal solutions in the experiment were supported by 0.01 mol/L NaNO_3_. The pH of the solution was adjusted to 5.0 by HNO_3_ and NaOH, the solution was shaken at 298 K and 220 rmp for 24 h. At a predetermined time, the mixed solution was filtered through a 0.45 μm filter membrane. The concentration of Pb was analyzed by a flame atomic absorption spectroscopy (TAS-990, Beijing Puxi, Beijing, China). The effect of pH was determined by adjusting the initial pH of Pb^2+^ solution (700 mg/L) from 2 to 5.5 with HNO_3_ and NaOH in the presence of nZVI@ATP (1 g/L). Pseudo-first-order kinetic model, pseudo-second-order kinetic model, and intra-particle diffusion model were used to analyze the adsorption kinetics, the optimal adsorption time was obtained, and the rate control steps of the process were determined. Anaerobic Pb^2+^ solution with an initial concentration of 550–950 mg/L at pH 5.0 ± 0.2, at 298 K, 308 K, and 318 K, the adsorption isotherm experiments of nZVI@ATP on Pb^2+^ were carried out. Langmuir, Freundlich and Temkin models were used to analyze the adsorption data. The influence of the coexistence of Na^+^, Ca^2+^, and Mg^2+^ on the adsorption of Pb^2+^ was studied by a single factor experiment with 0–10 mmol/L cations added into the initial concentration of 700 mg/L Pb^2+^ solution.

## 3. Results and Discussion

### 3.1. Characterizations of Materials

**Figure 1:**
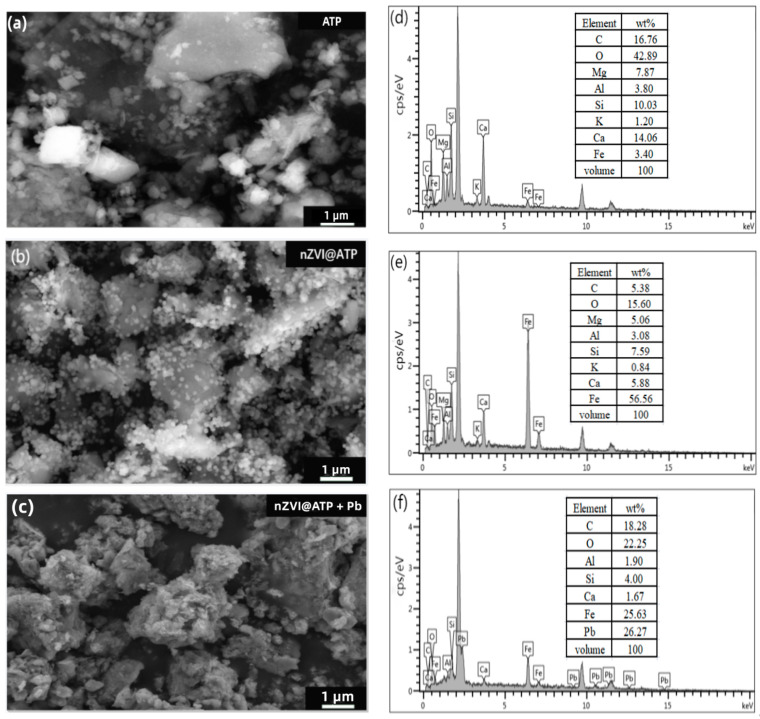
SEM image of (**a**) ATP, (**b**) nZVI@ATP, (**c**) nZVI@ATP + Pb; and EDS image of (**d**) ATP, (**e**) nZVI@ATP, (**f**) nZVI@ATP + Pb.

**Figure 2:**
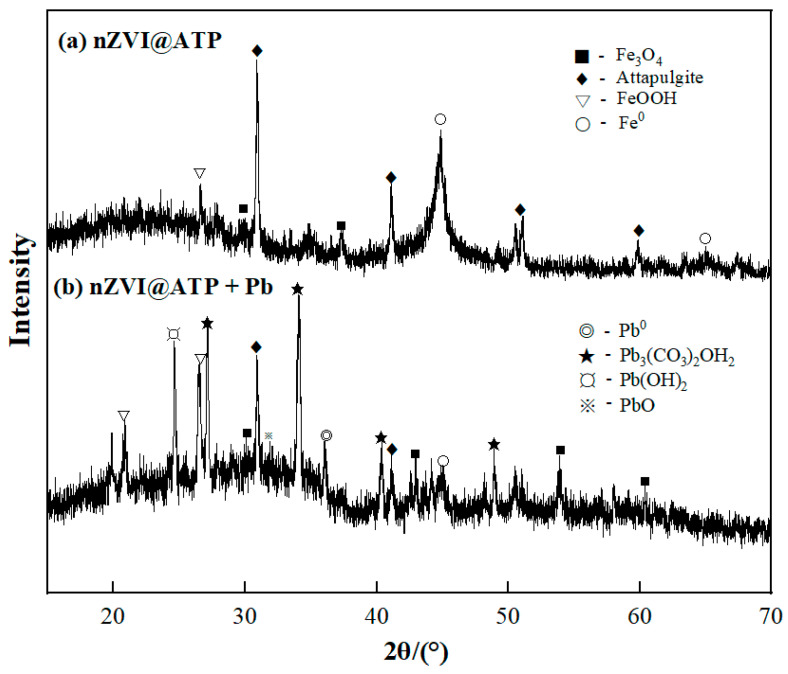
XRD patterns of nZVI@ATP, nZVI@ATP + Pb before (**a**) and after (**b**) adsorption.

According to the SEM images of nZVI@ATP before and after Pb^2+^ adsorption (Figure 1a–c), it can be found that spherical nZVI particles were dispersed and arranged on the surface of ATP before adsorption. After the adsorption of Pb^2+^, the morphology of nZVI@ATP changed greatly; the surface became rough, and nZVI particles could hardly be observed; instead, a large number of flocculent precipitates and some fine particles appeared, and the pore size became smaller, which was due to the chemical reaction between nZVI@ATP and Pb^2+^ and the reaction between Fe^0^ and water, resulting in the change of the microscopic morphology of nZVI@ATP. The EDS diagram in Figure 1d–f is the composition diagram of nZVI@ATP before and after the adsorption of Pb^2+^. It can be found that no Pb element was present in the diffraction peaks of nZVI@ATP before the reaction. After the reaction of nZVI@ATP with Pb^2+^, multiple diffraction peaks of the Pb element appeared, and the mass percentage reached 26.27%, indicating that Pb^2+^ had been successfully adsorbed onto the surface of nZVI@ATP. The XRD patterns of nZVI@ATP before and after Pb^2+^ adsorption are shown in Figure 2. In the XRD spectra of nZVI@ATP before the reaction, the peak value at 2θ = 44.86° represents the appearance of Fe^0^, indicating Fe^0^ formed on the ATP surface. The peak value at 2θ = 26.58 (FeOOH) indicates that iron oxide was also detected, meaning that Fe^0^ particles were partially oxidized during preparation or preservation. After adsorption, the characteristic peak of Fe^0^ (○) at 2θ = 44.86° weakens obviously, and the characteristic peak disappears at 2θ = 65°, indicating that Fe^0^ participates in the reaction, resulting in the reduction of its content. Meanwhile, the characteristic peak of FeOOH (▽) at 2θ = 26.58° significantly enhanced, and the characteristic peak of Fe_3_O_4_(■) appears at 2θ = 43.05° and 53.41°, both of which were transformed from Fe^0^. The sharp new diffraction peak at 2θ = 36.4° is attributed to the characteristic peak of Pb^0^ (◎), indicating that a reduction reaction occurred during the adsorption process, and part of Pb^2+^ is reduced to Pb^0^ by Fe^0^. The characteristic peaks at 2θ = 24.64° and 2θ = 31.9° correspond to the characteristic peaks of Pb(OH)_2_ (¤) and PbO (※), respectively, indicating that Fe^0^ will be oxidized or corroded to Fe^2+^ and further oxidized to Fe^3+^ during the adsorption process, and at the same time, OH^−^ was generated, and Pb(OH)_2_ precipitates were generated by the reaction with Pb^2+^, part of Pb(OH)_2_ was unstable in solution, and dehydration formed PbO [26]. The characteristic peaks at 2θ = 27.17°, 34.09°, and 40.34° are attributed to Pb_3_(CO_3_)_2_OH_2_(★), which is the product of the reaction of Pb(OH)_2_ with CO_3_^2−^ [26].

**Figure 3:**
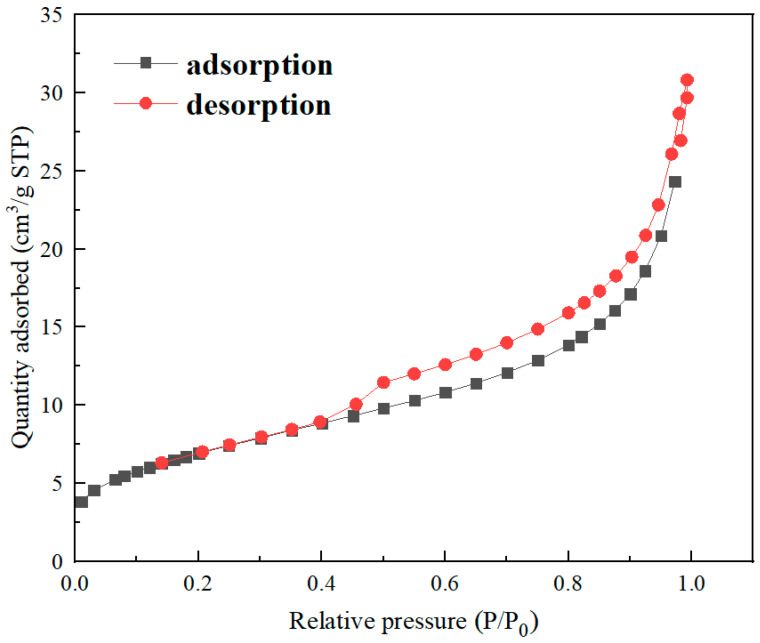
Nitrogen adsorption–desorption curve of nZVI@ATP.

The specific surface area of nZVI@ATP was measured by bet-N_2_ surface area analyzer, as shown in Table 1. The specific surface area (BET) and pore size of nZVI@ATP were 25.413 m^2^·g^−1^ and 7.229 nm, respectively, which is smaller than the BET of nZVI (20–60 m^2^/g) [27]. By analyzing the N_2_ adsorption–desorption isotherm curve in Figure 3, nZVI@ATP showed a type IV(a) isotherm (short platform, only inflection point). A comprehensive analysis of isotherms, BJH model, and T-plot micropore model showed that the pore structure of nZVI@ATP was mesoporous (2–50 nm).

### 3.2. Effect of Different Materials and Nanoscale Zero-Valent Iron Loading on Pb^2+^ Removal

After pickling, the removal rate of Pb^2+^ by ATP was 52.72%. In the acidification process, part of H^+^ was able to react with carbonate minerals in ATP, and part of H^+^ directly replaced cations in ATP. Soluble metal oxides and calcium carbonate impurities blocked the ATP pores and inside could be removed in large quantities, and the internal pores could be cleared by pickling, improving the pore size and porosity of ATP [28].

**Figure 4:**
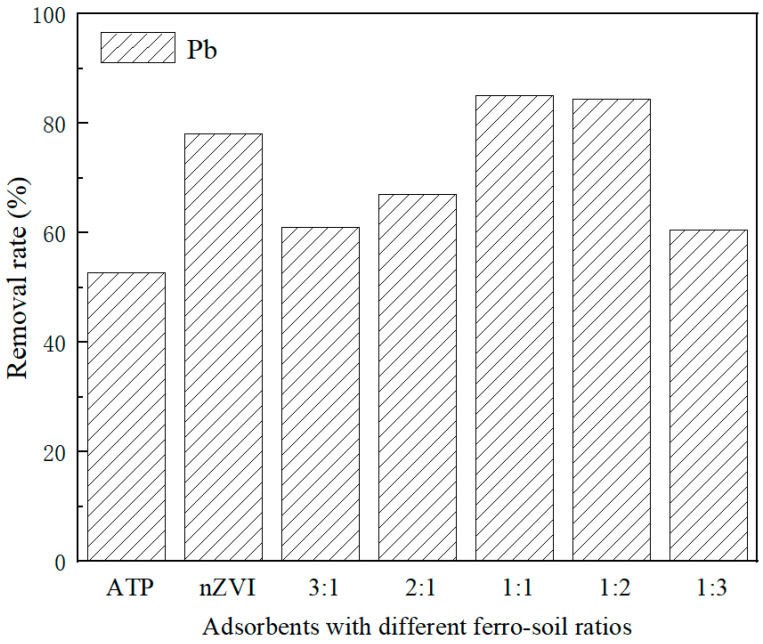
The removal rates of Pb^2+^ using different adsorbents.

The removal rate of Pb^2+^ by nZVI@ATP increased first and then decreased with decreasing Fe in the ratio Fe/ATP. As shown in Figure 4, the adsorption performance was best when Fe/ATP was 1:1 and 1:2, and the removal rate was 85.15% and 84.47%, respectively, which was also higher than that of pure nZVI (78.18%). Pure nZVI particles tend to agglomerate, which limits their contact opportunities with pollutants and cannot give full play to their adsorption properties, resulting in waste of adsorption sites. However, when loaded on ATP, the agglomeration phenomenon of nZVI could be improved due to the spatial barrier effect of ATP, and the removal rate could be increased. However, due to the limited spatial barrier effect of ATP, excessive nZVI would cover the surface of ATP and continue to aggregate into long chains, which inhibit the transfer process of heavy metal ions on the surface, resulting in a reduced removal rate. The high ratio of Fe/ATP was not conducive to the removal of heavy metals by nZVI@ATP. Since the removal rate of Pb^2+^ was not significantly different between Fe/ATP ratios of 1:1 and 1:2, the adsorption effect was better than that of pure nZVI, and when the ratio of Fe/ATP was 1:2, the iron content was only 1/3 of the iron in pure nZVI, the cost advantage was significant, and the adsorption characteristics and reaction mechanism were studied based on the subsequent experiments. Table 2 compares the adsorption performance of different adsorbents. The adsorption performance of nZVI@ATP on Pb^2+^ is higher than that of the other adsorbents mentioned above. Therefore, it can be seen that nZVI@ATP has important potential in removing Pb^2+^ from water.

### 3.3. Sorption Kinetics and Isotherms (Adsorption Properties)

#### 3.3.1. Sorption Kinetics

**Figure 5:**
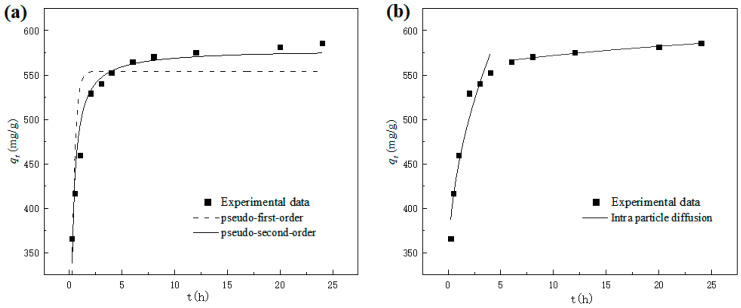
Adsorption kinetics for Pb^2+^ adsorption on nZVI@ATP: (**a**) kinetic model; (**b**) intra-particle diffusion model. Adsorption conditions: Pb^2+^ concentration = 700 mg/L, adsorption time = 0–24 h, pH = 5.0 ± 0.2, temperature = 298 K, adsorbent dose = 1.0 g/L, solution volume = 30 mL. The black squares in the figure stand for experimental data.

The influence of adsorption time on the adsorption capacity (*q_t_*) of nZVI@ATP for Pb^2+^ is shown in Figure 5. It is obvious that the adsorption capacity of nZVI@ATP on Pb^2+^ increases rapidly within 0–2 h and can reach more than 90% of the equilibrium adsorption capacity. After 2 h reaction, the growth rate of adsorption capacity slowed down until the adsorption equilibrium was reached (about 12 h). This is because at the initial stage of adsorption, a large number of vacant sites on the surface of nZVI@ATP are conducive to the adsorption of Pb^2+^, and the mass transfer force generated under the concentration difference of Pb^2+^ promotes the rapid diffusion of Pb^2+^ to nZVI@ATP, which has a good chance of being attached to the adsorption site. As the reaction progresses, the number of vacant adsorption sites on the surface of nZVI@ATP gradually decreases, and the mass transfer driving force caused by the concentration difference of Pb^2+^ decreases. Pb^2+^ must overcome greater resistance to enter the interior of nZVI@ATP, and combined with the adsorption site, results in a decrease in the adsorption rate, an increase in adsorption capacity, and gradual saturation.

The dynamic model was used to fit the experimental data, and the results are shown in Figure 5a. Table 3 presents the fitting parameters. The pseudo-second-order kinetic fitting parameter *R*_2_ was 0.9614, much higher than the pseudo-first-order kinetic fitting curve *R*_1_, and the theoretical equilibrium adsorption capacity *q*_*m*2_ obtained by the model fitting was 578.77 mg/g, with a relative deviation of only 1.16% from the experimental results, indicating that the pseudo-second-order kinetic fitting model was more suitable for the nZVI@ATP’s fitting curve of Pb^2+^.

Pseudo-first-order kinetic, pseudo-second-order kinetic, and intra-particle diffusion models are as follows:

(2) $$dq_{t}/d_{t}=k_{1}/\left(q_{e}-q_{t}\right)$$

(3) $$dq_{t}/d_{t}=k_{2}/{\left(q_{e}-q_{t}\right)}^{2}$$

(4) $$q_{t}=k_{d}t^{1/2}+E_{i}$$

*q_t_*—amount of heavy metals adsorbed at any time *t* (h) (mg/g)

*q_e_*—amount of heavy metals adsorbed at equilibrium (mg/g)

*k*_1_—pseudo-first-order rate constant (g/(mg·h))

*k*_2_—pseudo-second-order rate constant (g/(mg·h))

*k_d_*—intra-particle diffusion rate constant (mg/(g·h^1/2^))

*E_i_*—intercept (mmol/g)

The adsorption process of nZVI@ATP on Pb^2+^ was similar to that of nZVI@ATP on Pb^2+^. The fitting results of the intra-particle diffusion model are shown in Figure 5b, and the fitting parameters are shown in Table 4. In the model, the whole adsorption process could be divided into two stages (surface adsorption stage and intra-particle diffusion stage), *R*_1_^2^ < *R*_2_^2^, the intra-particle diffusion stage fitted well, and the intra-particle diffusion stage was the main stage of nZVI@ATP adsorption of Pb^2+^. *k_d_*_1_ was much larger than *k_d_*_2_, *E*_1_ < *E*_2_, and both were not 0, reflecting that the boundary layer at the diffusion stage was larger and the mass transfer resistance was larger. In terms of the rate of the entire adsorption process, the diffusion rate within the particle had a greater influence, but it was not the only speed control step.

#### 3.3.2. Adsorption Isotherms and Thermodynamic

**Figure 6:**
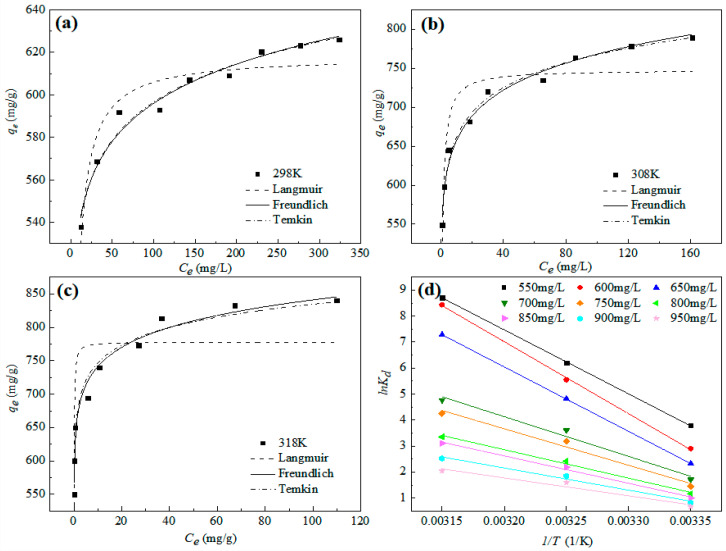
Adsorption isotherms and fitted models of nZVI@ATP for the removal of Pb^2+^: (**a**) 298 K, (**b**) 308 K, (**c**) 318 K, (**d**) Van t’ Hoff curve. Adsorption conditions: Pb^2+^ concentration = 50–450 mg/L, adsorption time = 24 h, pH = 5.0 ± 0.2, temperature = 298–318 K, adsorbent dose = 1.0 g/L, solution volume = 30 mL.

Figure 6 shows the fitting curves of the Langmuir, Freundlich, and Temkin isothermal adsorption models for the adsorption of Pb^2+^ by nZVI@ATP at different initial concentrations, and the results are listed in Table 5. The Freundlich isothermal adsorption model has the largest correlation coefficient, followed by Temkin isothermal adsorption model, and Langmuir isothermal adsorption model has the worst fitting result, indicating that the adsorption of Pb^2+^ on nZVI@ATP could be described well by the Freundlich model. During the adsorption process, nZVI@ATP had multiple adsorption sites on its surface. The adsorption of Pb^2+^ was characterized by multilayer adsorption, and the adsorption strength of 1/*n* was between 0.0440 and 0.0680, indicating that the adsorption of Pb^2+^ by nZVI@ATP was easy to carry out.

Langmuir, Freundlich, and Temkin isothermal adsorption equations are shown in (5), (6), and (7), respectively.

(5) $$q_{e}=\left(q_{m}K_{L}C_{e}\right)/\left(1+K_{L}C_{e}\right)$$

(6) qe=KFCe1n

(7) $$q_{e}=AlnK_{t}C_{e}$$

*q_e_*—amount of heavy metals adsorbed at equilibrium (mg/g);

*q_m_*—maximum adsorbed capacity (mg/g);

*K_L_*—Langmuir constant indicating the affinity of the binding sites for the heavy metal ions (L/mg);

*K_F_*—Freundlich adsorption coefficient (L/mg);

1n—adsorption intensity (0.1 < nf < 1);

*A*, *K_t_*—Temkin adsorption coefficient.

The thermodynamic formula of adsorption is as follows:

(8) $$\Delta G^{0}=-RTlnK_{d}$$

(9) $$\Delta G^{0}=\Delta H^{0}-T\Delta S^{0}$$

(10) $$lnK_{d}=\Delta S^{0}/R-\Delta H^{0}/RT$$

*R*—the gas constant, 8.314 J/(mol K);

*T*—the absolute temperature, K;

Δ*G^o^*—Gibbs free energy change, kJ/mol;

Δ*H^o^*—enthalpy change, kJ/mol;

Δ*S^o^*—entropy change, J/(mol K);

*K_d_*—the distribution coefficient of adsorption(mg/L).

The equation for calculating the apparent adsorption equilibrium constant *K_d_* (mL/g)

(11) $$K_{d}=q_{e}/C_{e}$$

*K_d_*—the distribution coefficient of adsorption (mL/g);

*q_e_*—amount of heavy metals adsorbed at equilibrium (mg/g);

*C_e_*—the remained concentration (mg/L).

According to Equation (11), the adsorption equilibrium constant *K_d_* at different initial concentrations and temperatures was calculated, and then linear fitting was carried out with 1/*T* as abscissa and ln*K_d_* as abscissa. The fitting results are shown in Figure 6d. The corresponding entropy change ∆*S*_0_ and enthalpy change ∆*H*_0_ were calculated according to the linear intercept and slope obtained by fitting, and the Gibbs free energy change ∆*G*^0^ was calculated by Equation (8) to discuss the influence of temperature on the adsorption process. Thermodynamic parameters are shown in Table 6.

As can be seen from Table 6, ∆*G*^0^ of nZVI@ATP’s adsorption of Pb^2+^ at different initial concentrations at different temperatures were all negative values, and the absolute value of ∆*G*^0^ increases with increasing temperature, indicating that the adsorption of Pb^2+^ by nZVI@ATP was spontaneous, and increases spontaneously with the increase in temperature, which promoted the adsorption reaction. It can provide more energy for Pb^2+^ to overcome the resistance caused by the diffusion electric double layer, and then be adsorbed on nZVI@ATP. ∆*S*^0^ and ∆*H*^0^ were both positive, indicating that the adsorption of nZVI@ATP to Pb^2+^ was an endothermic reaction with an increasing degree disorder at higher temperatures. Combined with the value of ∆*H*_0_ in Table 6, it can be concluded that the adsorption of Pb^2+^ by nZVI@ATP was mainly caused by chemical bond force, which again confirms the fitting results of the kinetic model.

### 3.4. Effects of Solution pH on Pb^2+^ Removal Capacity

The pH value of the solution is a crucial factor for the adsorbent to remove Pb^2+^. At low pH, Pb mainly existed in the free state of Pb^2+^. When pH approaches 6, the concentration of hydroxyl (–OH) in the solution increases, leading to the precipitation of Pb(OH)_2_ on the surface of nZVI@ATP, affect the actual adsorption effect. Therefore, the initial pH value of the solution was set in the range of 2–5.5 when the adsorption performance of Pb^2+^ was studied in this experiment [33].

**Figure 7:**
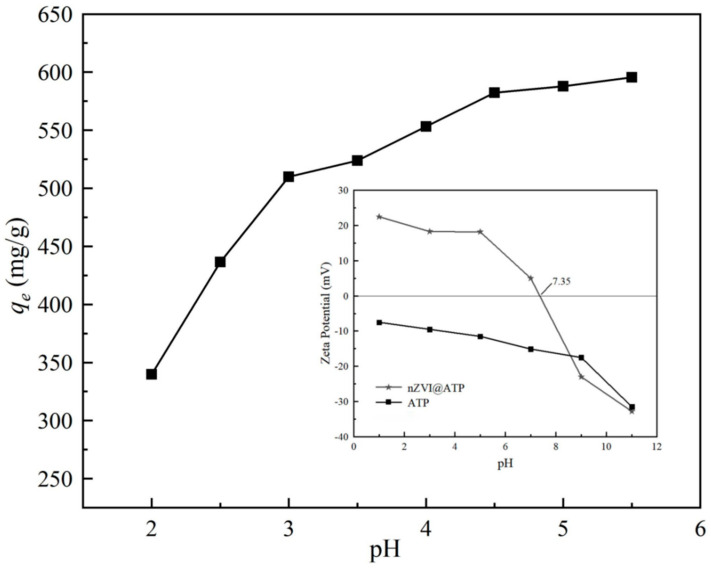
Effects of initial pH on the adsorption capacity of nZVI@ ATP for the removal of Pb^2+^. Adsorption conditions: Pb^2+^ concentration = 700 mg/L, adsorption time = 24 h, pH = 2.0–6.0 ± 0.2, temperature = 298 K, adsorbent dose = 1.0 g/L, solution volume = 30 mL. The inset in the figure caption shows the Zeta potential of ATP and nZVI@ATP as a function of pH; The black squares in the figure stand for experimental data.

Figure 7 shows the change of adsorption capacity *q_e_* of nZVI@ATP to Pb^2+^ with pH. As can be seen from the figure, the adsorption capacity of nZVI@ATP on Pb^2+^ increased with increasing pH, and the adsorption trend was consistent with previous research results [34]. The adsorption capacity of the solution increased from 339.70 mg/g to 595. 50 mg/g in the pH range of 2–5.5. When pH < 3.0, the adsorption rate was less than 50%, then with the increase in pH, the adsorption rate increased to more than 85%, it can be seen that the solution pH has a direct impact on the adsorption of Pb^2+^ by nZVI@ATP, where strong acidic conditions are not conducive to the adsorption of Pb^2+^ by nZVI@ATP. The main reason is that under strong acid conditions, a large amount of H^+^ in the solution would compete with Pb^2+^ with a positive charge for vacant adsorption sites on nZVI@ATP, reducing the adsorption efficiency of nZVI@ATP. In addition, under strong acidic conditions, nano zero-valent iron would be rapidly corroded, affecting its reaction activity, resulting in poor adsorption performance. With increasing pH, the competitive effect decreased and the adsorption capacity of Pb^2+^ increased. In addition, the oxidation of Fe^0^ under weakly acidic conditions would generate a large amount of OH^−^, improve the pH value of the solution, and promote the chemical precipitation of Pb^2+^ to be removed. This conclusion was confirmed in the subsequent XRD.

### 3.5. Influence of Coexisting Cations

**Figure 8:**
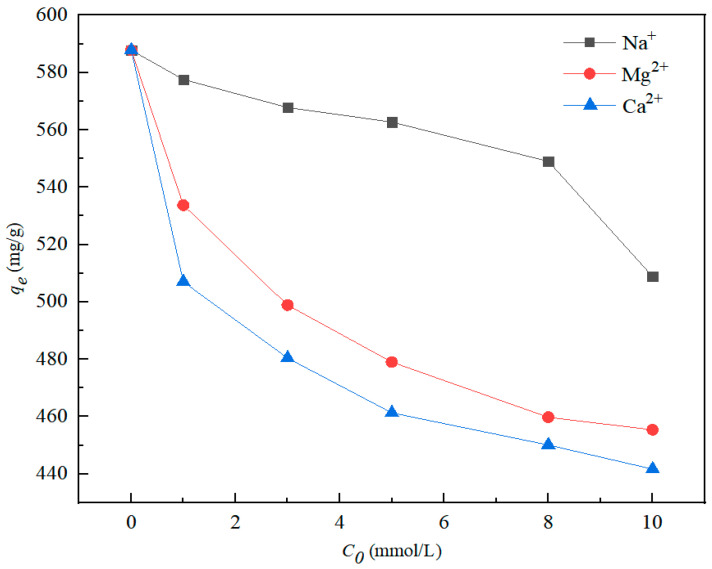
Removal efficiency of coexisting Pb^2+^ from water with different coexisting Na^+^ (0–10 mmol/L), Mg^2+^(0–10 mmol/L) and Ca^2+^(0–10 mmol/L). Adsorption conditions: Pb^2+^ concentration = 700 mg/L, adsorption time = 24 h, pH = 5.0 ± 0.2, temperature = 298 K, adsorbent dose = 1.0 g/L, solution volume = 30 mL.

Figure 8 shows the effects of three coexisting cations on nZVI@ATP adsorption of Pb^2+^. The presence of Na^+^, Mg^2+^, and Ca^2+^ could hinder the adsorption process of nZVI@ATP to Pb^2+^. When the ion concentration increased from 0 to 10 mmol, the adsorption capacity of nZVI@ATP for Pb^2+^ decreased from 587.8 mg/g to 508.85 mg/g (Na^+^), 455.40 mg/g (Mg^2+^) and 441.70 mg/g (Ca^2+^), respectively. The result shows that the adsorption of Pb^2+^ by nZVI@ATP decreased by 13.43% (Na^+^), 22.52% (Mg^2+^) and 24.86% (Ca^2+^), respectively. The three coexisting cations occupied the adsorption sites on nZVI@ATP and competed with Pb^2+^, reducing the adsorption efficiency of nZVI@ATP for Pb^2+^.

### 3.6. Pb^2+^ Removal of Mechanisms by nZVI@ATP

**Figure 9:**
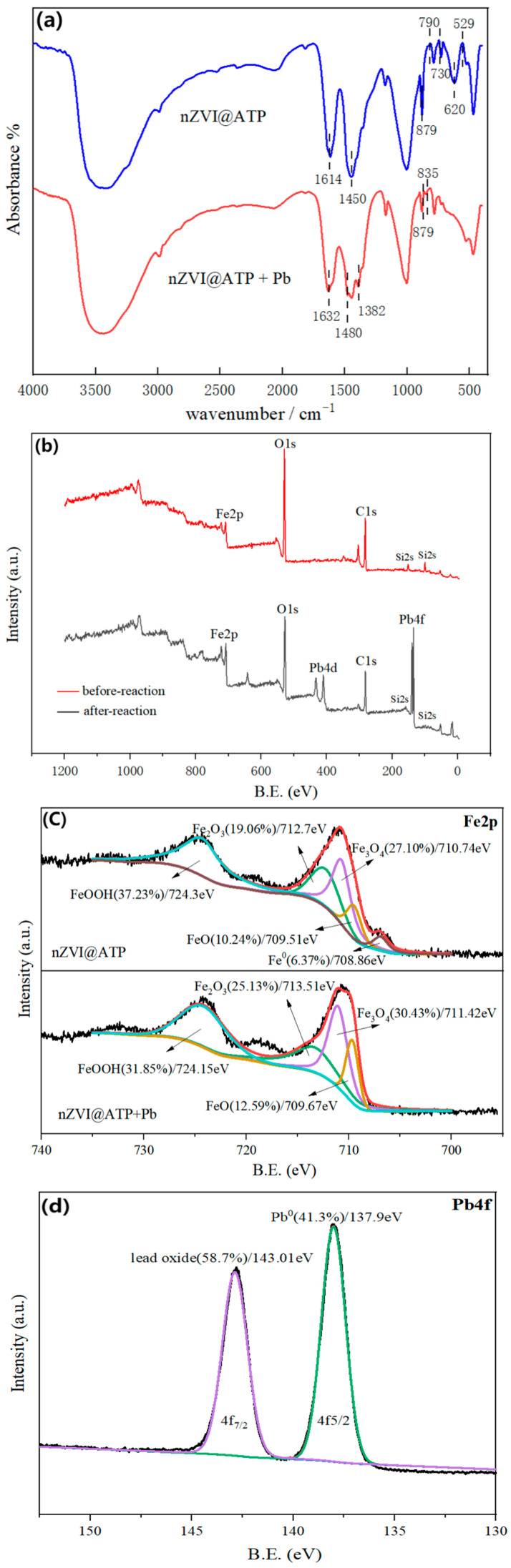
(**a**) FT-IR survey spectra of ATP before and after adsorption. (**b**) XPS survey spectra of nZVI@ATP before and after adsorption. (**c**) High-resolution XPS scan spectra over Fe2p of nZVI@ATP before and after adsorption. (**d**) High-resolution XPS scan spectra over Pb4f of nZVI@ATP.

FT-IR diagram of nZVI@ATP before and after Pb^2+^ adsorption (Figure 9a) shows that the hydroxyl peak in nZVI@ATP spectrum diagram with 3404 cm^−1^ was wide, and the hydroxyl peak strength increased and order degree became high, mainly due to the formation of hydroxyl oxide layer on the surface of nZVI particles. The peak of nZVI@ATP at 1614 cm^−1^ was the stretching vibration peak of H–O–H in the moisture substructure, the sharp and strong peak between 1211 and 1543 cm^−1^ was the combination peak of Fe–OH and CO_3_^2−^, and the new characteristic peak near 620 cm^−1^ corresponded to the lattice vibration peak of Fe–O in FeOOH [31,35]. This also indicates that, due to the high reactivity of nZVI, FeOOH was partially oxidized during the preparation or transfer process, which indirectly proved the successful modification of ATP by nZVI [36]. Several absorption peaks changed significantly in shape and strength before and after adsorption: the vibration peak of –OH at 1614 cm^−1^ shifted to 1632 cm^−1^, and the peak strength changed, indicating that –OH was involved in the adsorption reaction. The vibration peak at 1450 cm^−1^ was divided into two vibration peaks, among which the peak about 1382 cm^−1^ corresponded to the vibration peak of Fe–OH. The stretching vibration peak of Fe–OH was added at 835 cm^−1^, the characteristic peak of FeOOH previously located at 790 cm^−1^ shifted to 778 cm^−1^, and the weak peak of Fe–O decreased in the range of 730–470 cm^−1^, confirming the successful attachment of Fe^3+^ to its surface through its oxygen-containing group [31]. The formation of metal oxides on the surface of nZVI@ATP was confirmed, and the stretching vibration peak of CO_3_^2−^ at 730 cm^−1^ was significantly weakened, confirming again that CO_3_^2−^ was involved in the reaction [36].

XPS was used to analyze the valence state information and elemental composition of nZVI@ATP surface elements, as shown in Figure 9b. Before the reaction, the elemental composition was mainly Fe, O, C and Si, as shown in Figure 9b. After the reaction, the characteristic peak of Pb4f was detected at the position of 138.1 eV. This indicates that Pb^2+^ was successfully adsorbed on the surface of nZVI@ATP. As can be seen in Figure 9c, Fe^0^ appeared at the binding energy of 708.86 eV before nZVI@ATP reaction, which confirmed the presence of metallic iron in nZVI@ATP, indicating that nZVI was successfully loaded on ATP. nZVI@ATP corresponded to Fe^2+^ and Fe^3+^ at the positions of 710.74, 712.7, 709.51, and 724.3 eV before adsorption, which was due to iron oxides (2p3/2 and 2p1/2) formed by oxidation of nZVI surface (Fe–O) [37]. The characteristic peak of nZVI@ATP disappeared after adsorption, indicating that in the adsorption process, Fe^0^ participated in the reaction and converted it into iron oxide. Figure 9c shows the relative contents of different iron oxides. The relative contents of Fe_2_O_3_, FeO and Fe_3_O_4_ all increased after reaction, indicating that more iron oxides were generated during the reaction process, while the relative content of FeOOH decreased, indicating that it participated in the reaction of removing Pb^2+^. The Pb4f peak fitting diagram of nZVI@ATP is shown in Figure 9d, where 143.01 eV and 137.9 eV represented Pb4f7/2 and Pb4f5/2 respectively, which may be attributed to the formation of PbO, Pb(OH)_2_ and Pb^0^ [38]. The process of nZVI@ATP removing Pb^2+^ includes adsorption and reduction, that is, nZVI@ATP can physically adsorb Pb^2+^ in the solution, and then Fe^0^ in nZVI@ATP provides electrons to reduce Pb^2+^ to Pb^0^ [39]. Through XPS analysis, 41.3% of Pb^2+^ became Pb^0^, and the reaction equation was as follows: Fe^0^ + Pb^2+^→Fe^2+^ + Pb^0^.

## 4. Conclusions

nZVI@ATP has excellent adsorption performance for Pb^2+^, and when the ratio of iron to soil was 1:2, the removal rate of Pb solution with the initial concentration of 700 mg/L could reach 84.47% by nZVI@ATP, and the adsorption capacity was 591.29 mg/g. Through characterization analysis, it was found that nZVI presented regular smooth spherical particles with a particle size of about 50–100 nm. After loading on ATP, spherical nZVI particles were uniformly dispersed on the surface and interspaces of ATP, and the chain accumulation phenomenon was significantly weakened. The crystallinity of Fe^0^ in nZVI@ATP was low, some Fe^0^ was oxidized, and Fe_3_O_4_, FeOOH, and other substances were generated on the surface. The adsorption process of Pb^2+^ by nZVI@ATP conformed to the pseudo-second-order kinetic model, which indicates that the adsorption process was mainly chemical adsorption, and the speed control steps were composed of liquid film diffusion and intra-particle diffusion. The isothermal adsorption model was more suitable for the Freundlich isothermal adsorption model, which mainly consists of uneven surface adsorption. The adsorption process was a spontaneous endothermic reaction. The adsorption of nZVI@ATP to Pb^2+^ increased with the increase in pH in the range of 2–5.5. The presence of Na^+^, Mg^2+^, and Ca^2+^ could inhibit the removal of Pb^2+^, and the concentration of coexisting ions was inversely proportional to the adsorption capacity, among which Ca^2+^ has the strongest inhibition. The adsorption mechanism of nZVI@ATP to Pb^2+^ was mainly chemical adsorption. The removal of Pb^2+^ by nZVI@ATP would complex with the iron (hydrogen) oxide shell and precipitate Pb(OH)_2_ [38], PbO, and Pb_3_(CO_3_)_2_OH_2_ [31], and would also be reduced to Pb^0^ by the Fe^0^ core of nZVI.

## Figures and Tables

**Table 1:** Pore structure parameter of nZVI@ATP.

Sample	nZVI@ATP
BET Surface area (m^2^/g)	25.413
BJH Adsorption cumulative surface area of pores (m^2^/g)	19.993
Single point adsorption total pore volume of pores (cm^3^/g)	4.593 × 10^−2^
BJH Adsorption cumulative volume of pores (cm^3^/g)	4.391 × 10^−2^
Adsorption average pore width (4V/A by BET) (nm)	7.229

**Table 2:** Comparison of Pb^2+^ adsorption performance under the action of different adsorbents.

Sample	Carrier	*C*_0_ (mg/L)	*T* (K)	pH	Dosage (g/L)	*Q_t_* (mg/g)	Ref.
Zeolite and nanoscale zero-valent iron	Zeolite	100	308	4	1	96.2	[29]
Kaolin supported nZVI	Kaolin	500	303.15	*	5	440.5	[30]
nZVI-HCS	Hydrophilic corn stalk	50	298	7	0.25	195.1	[31]
Montmorillonite-supported nZVI	Montmorillonite	50	298	5	5	10.65	[32]
nZVI@ATP	ATP	700	298	5	1	578.77	This work

Note: * indicates that the initial pH of the solution has not been adjusted.

**Table 3:** Kinetics adsorption parameters of Pb^2+^ on nZVI@ATP.

*C*_0_	Pseudo-First-Order Kinetic Model			Pseudo-Second-Order Kinetic Model		
	*q_m_*_1_	*k*_1_	*R*_1_^2^	*q*_*m*2_	*k*_2_	*R*_2_^2^
700 mg/L	554.0631	3.3173	0.7701	578.7727	0.0098	0.9614

Note: *q*_*m*1_ (mg/g) is the theoretical adsorption capacity obtained by fitting the pseudo-first-order kinetic model; *q*_*m*2_ (mg/g) is the theoretical adsorption capacity obtained by fitting the pseudo-second-order kinetic model; *k*_1_ (g/(mg·h)) is the pseudo-first-order rate constant; *k*_2_ (g/(mg·h)) is the pseudo-second-order rate constant.

**Table 4:** Intra-particle diffusion parameters of Pb^2+^ on nZVI@ATP.

*C*_0_	*k*_*d*1_	*E*_1_	*R* ^2^	*k*_*d*2_	*E*_2_	*R* ^2^
700 mg/L	124.4606	324.7125	0.9356	7.8813	546.9407	0.9761

Note: *C*_0_ (mg/L) is the initial concentration of heavy metals; *K_di_* (mg/(g·h^1/2^)) is the intra-particle diffusion rate constant; *E_i_* (mmol/g) is the intercept, *i* represents phase *i*, *i* = 1 or 2.

**Table 5:** Langmuir, Freundlich and Temkin adsorption isotherm parameters for Pb^2+^ on nZVI@ATP.

*T*/K	Langmuir			Freundlich			Temkin		
	*q_m_*	*K_L_*	*R* ^2^	*K_F_*	1n	*R* ^2^	*A*	*K_t_*	*R* ^2^
298	618.1546	0.4935	0.8909	486.6824	0.0440	0.9730	25.8522	1.0512 × 10^8^	0.9759
308	747.8679	2.0096	0.8456	561.5242	0.0680	0.9842	46.1529	1.6551 × 10^5^	0.9887
318	777.6810	24.2099	0.7725	653.3360	0.0548	0.9710	38.0106	3.4127 × 10^7^	0.9678

Note: *q_m_* (mg/g) is the maximum adsorbed capacity; *K_L_* (L/mg) is the Langmuir constant indicating the affinity of the binding sites for the heavy metal ions; *K_F_* (L/mg) is the Freundlich adsorption coefficient; 1/*n* is the adsorption intensity; *A*, *K_t_* is the Temkin adsorption coefficient.

**Table 6:** Thermodynamic parameters for Pb^2+^ on nZVI@ATP.

*K_d_*	*C*_0_ (mg/L)	Δ*H^o^* (kJ/mol)	Δ*S^o^* (J/(mol·K))	Δ*G^o^* (kJ/mol)		
				298 K	308 K	318 K
*K_d_* = *q_e_*/*C_e_* (mL/g)	550	204.876	717.688	−8.995	−16.172	−23.349
	600	230.271	795.176	−6.692	−14.643	−22.595
	650	206.058	709.607	−5.405	−12.501	−19.597
	700	127.144	441.172	−4.325	−8.737	−13.149
	750	116.786	404.169	−3.657	−7.698	−11.740
	800	91.081	315.223	−2.856	−6.008	−9.160
	850	87.577	302.014	−2.423	−5.443	−8.463
	900	70.817	244.507	−2.046	−4.491	−6.936
	950	57.215	197.816	−1.734	−3.712	−5.691

Note: *K_d_* (mL/g) is the distribution coefficient of adsorption; *C*_0_ (mg/L) is the initial concentration of heavy metals; Δ*H^o^* (kJ/mol) is the enthalpy change; Δ*S^o^* (J/(mol·K)) is the entropy change; Δ*G^o^* (kJ/mol) is the Gibbs free energy change.
